# Disability and the Family in South Wales Coalfield Society, c.1920–1939

**DOI:** 10.1080/14631180.2017.1316030

**Published:** 2017-06-14

**Authors:** Ben Curtis, Steven Thompson

**Keywords:** disability, family, community, theory, south Wales

## Abstract

This article utilises the south Wales coalfield in the interwar period as a case study to illustrate the applicability of two sociological theories – family systems theory and the social ecology of the family – to impairment in the past. It demonstrates that a theoretically-informed approach can help to situate impairment in its particular contexts, most especially the family and the community, and give a better sense of the lived experience of disability. It also demonstrates the complexity of the experience of disability as the family and economic circumstances of each impaired individual varied and led to different forms of care-giving or the utilisation of different sources of support. The article also sheds further light on the ubiquity of disability as many families included a number of individuals with different impairments and this too had consequences for experiences and coping strategies.

In the study of history, it is sometimes the case that the utilisation of a methodology or theoretical perspective borrowed from another academic discipline can generate new research questions or help to illuminate a hitherto obscure historical issue or phenomenon. One potentially very promising area in this respect is the study of the social context of disability: this is a topic which has engaged the attention of disability historians, social theorists and psychologists, and disability studies scholars, all of whom have approached it from a range of perspectives and employing an array of different approaches. In this article, we discuss two overlapping theories which have their origins in sociological analysis and are utilised in contemporary social work – namely, family systems theory and the social ecology of the family – and consider the ways in which these can be applied to a particular historical context. As historians, we find that these sociological models suggest all manner of fruitful ways in which to think about disability in its everyday contexts in the past and thereby enrich the historiography with a more penetrating analysis of the lived experience of disability.

One of the main sociological approaches to questions of disability has been to examine the social systems within which a disabled individual operates. In the first instance, within a family, this has been analysed by family systems theory, which examines the principal variables in the different types and levels of interactions within families.^1^ Family systems theory was developed from the 1950s onwards as psychiatrists, therapists and theorists, primarily in the United States, shifted their attention from the individual as the ‘problem’ to the family unit as a whole and the interactions that occurred between its members. Crucial here was the work of Murray Bowen who conceptualised the family as an ‘emotionally governed system or unit’ in which changes in the functioning of one family member had an impact on functioning of other members of the family.^2^

At the same time, the family does not exist in isolation but instead operates within a broader context – this has been characterised by some sociologists as the ‘social ecology framework’.^3^ In the social ecology model, there are different levels of interaction, both within and between families, communities, organisations and society more broadly. These sociologically-informed theories have generally been constructed for use in social work in relation to a range of social issues, most prominently the health, welfare and well-being of children, and for the most part eschew any kind of historical perspective.^4^ Nevertheless, provided that they are interpreted appropriately and not superimposed as a rigid and prescriptive model, we believe that these sociological perspectives can help to provide a framework within which to analyse the complex and tangled nature of the reality of disability, both for the individual and the family. It is with this idea in mind that this essay attempts to conceptualise and understand the social ecology of disability in interwar south Wales coalfield society.

To some degree, many of these ideas and theories coincide with the ways in which historians have treated families in their work: historians have not been unaware that the individual does not exist in isolation from society but instead resides within overlapping and interconnected clusters of familial and social networks, themselves located within the broader socio-economic context. A great deal of work has been published in recent decades that sets out to broaden understanding of sickness and impairment in all their varied contexts. A key priority within this historiography of health, illness and well-being has been to identify the ‘locus of care’ – that is, the structures and agencies which assisted and cared for sick and impaired individuals. Within this work, the impact on families on the one hand and the decisions taken to seek assistance from different providers on the other have attracted increasing levels of attention in recent years.^5^ In work on the history of mental illness and learning disability, for example, historians such as David Wright, Cathy Smith and Steven Taylor have attempted to explore the emotional and practical impacts of such impairments on the families of sufferers and the considerations that led to decisions to seek assistance from outside the family unit, in these instances through the admission of the mentally ill individual into an asylum.^6^

Clearly, the concept of the ‘mixed economy of care’ is an important insight and is a recurrent motif in the work of many historians of welfare.^7^ The role of the family has been identified as a key factor: Lewis, for instance, has stated unambiguously that ‘the family has always been the main provider of welfare’.^8^ In contrast to this rather straightforward characterisation, Horden and Smith argue the case for the idea of ‘a complicated and shifting ‘mixed economy’ of care, in which the role of the immediate family may have been overestimated’.^9^ As far as disability is concerned, historians have produced a large number of well-researched and carefully observed studies of the history of disability and disabled individuals, but the purpose of this article is to suggest that an approach which is influenced by a more theoretical sociological perspective may help to provide a fuller understanding of the lived experience of disability. In attempting to sketch a framework which can be used to help to identify and examine the myriad of ways in which a disabled individual interacted with society within a particular socio-historical context, we believe that it is possible to construct theoretically-informed analyses which provide a concrete platform for comparative analysis and offer real explanatory insights, while at the same time remaining sensitive to the nuances of historical specificity.

Coal mines were an intrinsically dangerous place to work. Between 1885 and 1949, coal mining consistently accounted for about 25% of all occupation-related accident deaths in the United Kingdom, sometimes significantly more.^10^ As Benson observes, ‘between 1868 and 1919 a miner was killed every six hours, seriously injured every two hours and injured badly ... every two or three minutes’.^11^ Coal miners worked in an unpredictable environment where their bodies were directly exposed to a variety of hazards, ranging from gas and explosions to dust, rock falls and equipment failure. Physical impairment among women servicing the coal industry as wives and mothers was also unusually high.^12^ The coal industry exacted a fearsome toll in terms of injury, disability and occupational illness, on a literally industrial scale. Within this general picture of death and disabling injury, south Wales was the most dangerous coalfield in Britain.^13^ Between 1890 and 1939, south Wales accounted for an average of 25.14% of total British colliery deaths despite the region being home to a maximum of only 21.22% of the mining workforce (in 1913, the year of the greatest-ever output attained in the south Wales coalfield).^14^ Consequently, approximately one in sixteen of all occupational mortality in all industries across Britain between the late nineteenth century and the mid-twentieth century occurred in the south Wales coal industry. Non-fatal industrial injury was also similarly prevalent there. For the south Wales miners, the risk of death in the workplace was ever-present and the experience of serious and disabling injury was commonplace. The decision to focus this study on the interwar decades partly reflects the fact that this is the period for which the relevant extant archival evidence is richest. The period is also of interest because the dramatic decline in the fortunes of the south Wales coalfield in the interwar years made the socio-economic climate even more problematic for the many thousands of its disabled inhabitants and indeed as it revealed their living arrangements as a result of applications for relief and the resultant investigations into their means.

## Family systems theory and disability in the south Wales coalfield

The experience of disability was (and is) mediated by the specific family circumstances of the disabled individual and, we contend, family systems theory, which examines the principal variables in the different types and levels of interactions within families offers an analytical framework in which to consider such mediation. It also enriches any study of disability by its attention to the impact of impairment on other family members and the functioning of any family.^15^ According to family systems theory, there are four distinct subsystems within a family, which describe the various kinds of familial relationships. These are: marital; parental – between parent(s) and child(ren); sibling – between the family’s children; and extra-familial (extended family, friends, professionals, and so on). Each of these subsystems is further affected by two other key variables: family structure (for example, if there is only a single mother or father, as well as the number of children) and family life-cycle stage (for instance, where the parents are in terms of their relation to their working career; also, whether the family’s children are pre-school, of school age, in employment but still living at home, or have left home and started families of their own). Mishak, Seligman and Prezant observe that ‘[i]n terms of the effects a disability may have on family activity and functions, it is conceivable that the family’s self-identity will change, its earning capacity may be reduced, its recreational and social activities may be restricted, and career decisions may be affected.’^16^

In order to be more meaningful for our purposes, this sociological analysis needs to be contextualised within the socio-economic setting considered here – namely, the south Wales coalfield in the early twentieth century. Whilst it is misleading to overemphasise the usefulness of abstract aggregate concepts such as ‘the average family’ when considering the specific circumstances of a particular individual family, it is nevertheless worthwhile for us to bear in mind the broad demographic features of coalfield communities at this time – especially given that in certain respects they *did* differ from the average British picture as a whole, in ways which have a bearing on our analysis. The first question to consider is that of family size. The 1911 Census, for instance, shows that at this date the typical mining couple had 4.23 children, a number significantly above the national average of 3.53.^17^ This was partly due to the high wages that could be earned by young men in their early twenties in the coal industry that encouraged household formation at relatively earlier ages and which then resulted in higher fertility rates.^18^ These factors meant that female members were not required to enter the labour market to quite the same degree as in other regions, since men could reach their peak earning years in their early twenties, and greater pressure was placed on girls and women to fulfil domestic responsibilities in the home, in the form of child-care, cleaning, washing, and the preparation of food. This, together with the particularly gendered character of labour in the coal industry, meant that female economic activity levels were relatively low in south Wales.^19^ Across Britain as a whole in 1939, for instance, 39 women were employed for every 100 men; the ratio in industrial south Wales at that time was 16 to 100.^20^ Moreover, households often included a number of male breadwinners as sons followed their fathers into the pit, often from the age of fourteen onwards, in addition possibly to a lodger who was also a miner. Although statistics of this kind are not necessarily an accurate guide to understanding the particular circumstances of an individual miner and his family, they nevertheless do indicate in broad terms that mining families tended to be larger than the national average in the early twentieth century and also that, in south Wales, mining families tended to be dependent upon male ‘breadwinners’ to a greater extent than other occupational groups elsewhere in Britain.

Apart from the variety of family circumstances, it was also the case that the character and extent of disability varied greatly from one person to another, and this adds an extra level of complexity to our analysis, since the character and extent of impairment had consequences for the family’s ability to cope and the ways in which it functioned as a unit. Although disability manifested itself in a myriad of forms, the practical consequences of industrial injury or disease for mineworkers can perhaps be considered in a fairly small number of general categories. Major physical injuries (as opposed to comparatively minor flesh-wound injuries) tended to fall into one of two classifications: either severe, permanent injuries (such as head injuries, the loss of a limb or limbs, or paralysis), or significant but potentially non-permanent injuries (such as broken bones). The impact of industrial disease depended upon the properties of the disease itself. However, to take the two most common diseases, pneumoconiosis (a condition in which miners’ lungs became damaged and congested owing to inhalation of coal dust particles over a period of time) and miners’ nystagmus (a condition of involuntary oscillating eye movement, caused by working underground in poor lighting conditions): pneumoconiosis was a degenerative condition which could eventually become fatal, whereas nystagmus was serious but episodic in nature. Miners with nystagmus might feel their symptoms alleviated upon ceasing work underground, but would be likely to suffer a relapse if they returned to subterranean employment at any point subsequently. Finally, there were also a whole range of chronic but relatively minor ailments, such as hernias and rheumatism. Although rarely fatal, these would have been a source of continual pain and discomfort. Despite being less dramatic and conspicuous than other disabling conditions, the cumulative effect of this ‘wear and tear’ on miners’ bodies could still be debilitating and should not be underestimated; evidence from the south Wales coalfield shows that it was possible for some miners in their twenties to require admittance to the infirmary on account of the severity of their rheumatism.^21^ As Benson notes, ‘[e]ven if he should be lucky enough to avoid serious injury and crippling disease, the miner and those around him had to learn to live with his tiredness, his aches and pains, his ruptures and his rheumatism.’^22^

A further factor that worked to influence a family’s ability to cope with disability was the family life cycle and the point at which impairment occurred within it: there would have been different consequences for the family of a young miner (who had a family depending upon him) to become disabled as compared with an older miner (who perhaps did not and who could possibly have looked to grown-up children for support). This point can be illustrated with two examples from the records of the Bedwellty Poor Law Union’s Out Relief Advisory Committee. The first of these appears in February 1924, that of a thirty-one year old miner from Hollybush near Blackwood, who had ceased underground work at Markham Colliery owing to nystagmus, and who was married with eight dependent children aged between eleven years and six months. He had resumed light work at the colliery but consequently found that his compensation payments from the coal company had ceased.^23^ His comparative lack of family support resources stands in marked contrast to another case reported to the committee in December 1926. Here, a forty-eight year old former miner, from Aberbargoed, had sustained an injury to his head in 1915 while working as a collier and had not worked since. Although he had four dependent children, the household numbered eight people including four adults, with two of his sons being in paid employment and with a daughter staying at home to assist his wife with household duties.^24^ The experience and consequences of disability were quite different in each of the two cases and family members in each case would have been affected by, or might have ameliorated, the consequences differently.

The physical symptoms of industrially-induced disability were all too real for those miners who experienced them, but they were in some senses only the most obvious manifestation of a whole swathe of changes which were involuntarily visited upon a disabled miner and his family. The first point to be mentioned here is the impact of a miner’s disability upon his family’s economic situation. Essentially, any degree of disability led to a decline in the amount of income that a miner brought into the household. Although a system of compensation for serious industrial injuries existed following the enactment of the Workmen’s Compensation Act of 1897, even the full compensation payment (which stood at 35 shillings a week in the mid-1920s) would certainly have been less than a collier’s wages, which would generally have exceeded £2 a week at that time.^25^ The same point about a reduced level of income would also have applied to any disabled miners who were able to resume less physically arduous work elsewhere in the colliery – for example, working in the colliery lamp room. Even comparatively minor ailments, such as hernias and rheumatism, would have impacted materially on a miner’s income, given that most wages in the industry at that time were paid on a piecework basis according to the individual’s output. Sometimes, though, it was possible for a disabled miner to take up work outside of the coal industry or even set up his own small business: for example, a collier from Nantyglo who was injured in a pit accident in North Blaina Colliery in 1906 but who was subsequently able to establish himself as a confectioner.^26^

Apart from financial consequences, industrial disability could have far-reaching, complex psychological effects upon mineworkers. Disabled miners and their families had to deal with the psychological repercussions of the diminution or loss of household income, significant physical impairment or deterioration, and invariably, the deeper implications of dependency and loss of self-esteem; these, in turn, would quite possibly bring about changes in established family roles, which undoubtedly would have impacted upon intra-family relationships.^27^ These effects would have been exacerbated if disablement occurred at a relatively young age – an all too common occurrence. Disability diminished or removed the removed the ability of male workers to play the ‘breadwinner’ role for their families and also took away their physical strength and consequently their ability to perform ‘man’s work’. For instance, one miner with advanced pneumoconiosis noted that his wife had to shovel the house coal in his household as he was unable to do so, commenting that ‘When the winter period come I had to stop in because of the extreme cold, I couldn’t breathe … I move around quietly, there is no exertion now … I have had a bed now put in the parlour, these last two years to save me climbing the stairs … I haven’t been upstairs for two years’.^28^ In such a ‘masculine’ society as the south Wales coalfield in this period, the impact of this upon an individual’s sense of identity and self-esteem could indeed be profound. Disability would also have compounded this potential sense of emasculation by quite possibly necessitating the removal of an injured miner from a male-dominated work environment and relocating him instead within the ‘female’ domestic sphere. It should be noted, though, that in this respect some miners were able to adapt better to their changed circumstances than others. George Preece, for example, lost both his legs in an underground accident at Abercynon in 1909: he subsequently spent much of his time in the company of his cousin and her children and became skilled at crocheting.^29^ Furthermore, any miner who became severely disabled would have quite possibly also found himself unable to participate in his former pastimes and social events, some of the most popular of which (such as going to the pub or playing sports) were also very masculine-orientated, thereby further underlining this sense of loss of identity. As one disabled miner commented ‘a man’s life is not confined to his work. He has a social life, and the consequence of an accident like the loss of a leg or an arm or an eye, was with him, when he was trying to enjoy some social life and domestic life, and not simply that he couldn’t work. We’re not simply cogs in a wheel’.^30^ One extreme manifestation of this diminished ability to participate in social activities was noted by B. L. Coombes, a miner from Resolven who wrote extensively about the realities of day-to-day life in the south Wales coalfield in the 1930s and 1940s. Coombes observed that some former mineworkers in his village had become so debilitated by pneumoconiosis that they were no longer able to leave their homes, with their sole annual trip out-of-doors being to be helped out onto the pavement to watch the annual procession of chapel congregations as it passed by.^31^

Not only does impairment have practical, financial and social impacts on individuals and families, it also disrupts and alters the emotional functioning of the family, and, in this context, family systems theory is especially suggestive for historians of disability. Murray Bowen conceptualised the family as an emotional unit and, since that time, the emotional interactions between members of the family have been central to social work with families and the therapy provided. Family systems theory considers the tension or anxiety that occurs when a traumatic ‘stressor’, such as impairment, arises and the extent to which togetherness or differentiation occur as a result of the pressure. Togetherness derives from an emotional reaction to any situation that causes families to draw closer together for support but which can also impair their ability to arrive at rational, logical decisions, while differentiation increases the individuation of family members that can assist them to reason rationally, rather than emotionally, but can lead to fragmentation as individual members pull away from the family unit. It is believed that a correct balance between togetherness and differentiation leads to love, loyalty and support on the one hand but also personal responsibility and self-determination on the other, thereby equipping a family to cope with pressures more effectively.^32^ It is no easy task for historians to study the emotional interactions within families and the extent of togetherness or differentiation that occurred over time. This is particularly marked for historians of poor and working-class families since first-hand accounts are relatively rare while the types of institutional sources drawn upon here, whether Poor Law records or materials derived from voluntary associations, tended to prioritise household means, individual needs, and the forms of assistance to be granted rather than the internal dynamics of families as emotional units.^33^ More autobiographical sources, such as oral history, working-class autobiographies, and correspondence with authorities and institutions, will need to be utilised to get a sense of the emotional dynamics within families.

Notwithstanding the variety of ways in which disability could impact upon the lives of miners and their families outlined above, the model as sketched thus far is insufficiently complex. In practice, the arduous and hazardous nature of life and work in the south Wales coalfield in the early twentieth century meant that it was entirely possible for there to be several individuals with disabilities within a given family. This multiplicity of disabilities had profound and complicated consequences for the practical, financial and emotional functioning of the family. Taking the family as a whole, such disabling conditions need not necessarily have had any direct connection to work in the coal industry, but could have been the result of illnesses such as tuberculosis or various congenital impairments. In each case too, the timing and extent of the occurrence or emergence of each specific disability within a particular family group is also pertinent – both in terms of which condition came first and also whether each was a sudden or a gradual-onset development. These additional factors complicate the picture significantly, as the following representative examples illustrate. One commonplace situation of multiple disabilities within a given household would have been the disablement of both the main male ‘breadwinner’ and an adult child as a result of injury or accident. For instance, in a case from 1923, a forty-nine year old man from Pengam, suffering from ‘General Debility’, had a son in receipt of full-scale industrial compensation (of thirty-five shillings per week) living at home with him (in addition to his wife, two dependent children, another son aged sixteen years and in employment, as well as a further, married, son with his own dependent child).^34^

Another possibility was for there to be an industrially-acquired disability or injury as well as a congenital impairment within a given family. One example from the Bridgend Poor Law Union records from June 1929 is that of a twenty-one year old unmarried man from Aberkenfig, described as being a ‘cripple since birth’. This case demonstrates the significance of the above point about the timing of the occurrence or emergence of the various disabilities within a family: the case notes state that he ‘resides with his parents at the above address, the Father of the applicant not able to keep this son because he has been unemployed thro’ an accident at the Colliery on May 15th [1929]. Compensation to Father not yet received’.^35^ Here, the familial circumstances would have meant that the injury to the father would have been a particularly severe blow to them. In addition to the miners themselves, their dependent children could also be disabled to varying extents, either congenitally or via an injury. One example of the former, from 1935, is that of a seven-year old boy from Llangennech who required use of a ‘leg instrument’ (i.e. a calliper) and whose father was a fitter at Morlais Colliery who ‘earns 25/ in a good week, [and] has [a] disablement pension’.^36^ An example of the latter, also from 1935, is that of an eleven-year old girl from Cwmllynfell who lost an eye in an accident in August that year, whose father was employed as a lampman at Gwaun-cae-Gurwen Colliery and also in receipt of partial-rate industrial-injury compensation.^37^

In some instances, a family in which there were a number of dependent children could have both parents disabled or incapacitated to varying degrees. In one case from 1926, the Bedwellty Poor Law Union records note a family from Nantyglo in which the father (aged forty-four) was suffering from a hernia and the ‘effect of an old injury to the back and is of a nervous disposition’ and had not worked since 1921 (although it is not clear to what extent this was as a result of his injuries). The mother, aged forty-two, ‘is suffering from a severe form of Spinal Curvature, Asthma, Bronchitis, & collapse of Lower Right Lung’; she is described as being ‘in a weak condition’ and ‘not capable of performing a day’s washing’. They had four dependent children.^38^ In practice, it was possible for a whole extended family household to be affected by multiple disabilities and misfortunes. This can be seen quite clearly in the case of a family from the practically mono-industrial mining town of Blaengarw, whose circumstances were examined by local Poor Law administrators in 1928, when they applied for relief. The applicant himself had a medical certificate proving he was ‘physically incapable of following his employment’; he resided with his niece, a widow with two children dependent, who was in receipt of weekly compensation on account of her husband’s death; the applicant’s nephew also lived with them and ‘earns an average wage of £2: 5: 0: weekly, but works very irregular owing to illhealth [*sic*]’.^39^ In such instances, the changing dynamics between these different family members, with their varied impairments, would have placed severe stress on the family unit and tested its resilience and its ability to find homeostasis.

The particular position within his/her family of an individual who became disabled is another important consideration, as this had a bearing on how the specific day-to-day practicalities changed for that family. As family systems theory posits, ‘[a] disability in the family has implications for the functions family members assume. Considerable interdependence within the family and its extra-familial network is required so the necessary functions are performed for survival.’^40^ Whilst this is the case in general terms, a further complicating factor in this respect is the broader socio-economic and cultural frameworks within which the family operates. From the perspective of the south Wales coalfield in the early twentieth century, what this meant was that expectations of individuals’ particular roles within the family were strongly influenced along highly gendered lines, with men (and their sons, once they were old enough) going to work (typically in the mines) and women and girls tending to remain in domestic-orientated roles, particularly once women had married. Although it is something of an oversimplification, what this would have tended to mean in south Wales coalfield society in this period is that the primary familial effect of impairments to male wage-earners would have adversely affected household income; impairments to adult and adolescent females would have adversely affected domestic functioning; and impairments to dependent children would not *directly* affect either household income nor domestic functioning but would certainly have stretched family resources (financial, domestic, and emotional).

In each case, of course, the way in which this generalised tendency impacted upon a particular family was mediated by all its various specific characteristics, in terms of personnel, life-cycle stage, and so on. A disabling injury for a miner would have been quite likely to impact upon the range of economic options open to other members of his family, particularly adolescent girls and young women still living in the family home.^41^ One example of this, reported in 1926, is that of a miner from Blaina, who had been out of work since suffering a fractured spine in 1921. He had a wife and three dependent children; he also had a sixteen-year old daughter who was consequently ‘unable to take up domestic service. Needed at home to assist to nurse her father’.^42^ In the language of family systems theory, an older child who takes on caring responsibilities for younger siblings is inducted into the ‘parental subsystem’ (i.e. that part of the family responsible for raising children), with all the emotional and practical consequences that that brings; in instances where children came to care for parents, on the other hand, as was relatively common in these industrial communities in the past, the changes in the emotional functioning of the family were even greater and the consequences for the individual child that much more significant.^43^

Conversely, some families, when faced with the adverse economic consequences of disability, made the decision to limit family size and have fewer children; the existing children in such families were also forced to enter the world of work at younger ages, rather than remain in education or help in the home, with the diminished life chances that that brought them.^44^ In several respects, the most significant impact upon the ‘locus of care’ within a family was felt when it was the wife/mother within a family who was ill or disabled. A combination of socio-economic necessity, the arduousness of mine work, and cultural expectations would have militated against miners taking up the primary domestic role in such circumstances, although White and Williams have suggested that behind closed doors men perhaps participated more in domestic tasks than was previously thought and played at least some role in care-giving.^45^ One such example is provided by a case relieved by the Swansea Hospital Ladies’ Samaritan Fund in 1911, in which a collier, who had had his hand amputated as a result of blood poisoning, had no wife to care for his three young children while he spent a period of time at a convalescent home, and was forced to turn to his brother for assistance, who was ‘also a working man’; presumably the father assumed responsibility for the care of the children upon his return.^46^ Generally, though, unless they had a daughter or daughters capable of taking up the household responsibilities, families where the mother became disabled or was absent for whatever reason would have been forced to rely upon extra-familial assistance – even if this meant paid help which stretched already limited financial resources. One example, from 1928, is that of a woman from Loughor, the mother of ‘3 little children’ including ten-month-old twins, whose husband was employed as a colliery lampman. She required an artificial leg and consequently the family ‘have to pay a girl to work in the house as [she] cannot do it till she gets a leg’.^47^ The employment of a young girl to assist with domestic tasks in households with a disabled individual, whether the husband or the wife, was relatively common.^48^

In most instances, however, it was the women of households who were the main care-givers in the event of disability. This, of course, was entirely consistent with dominant gender ideals and women were quite used to the considerable tasks that household management entailed. Nevertheless, disablement of the male worker created a considerable amount of additional work, in addition to tasks that would otherwise have been carried out by the man, and it is likely that the physical and emotional burdens placed upon women were significant and probably contributed to the poor standards of health and well-being of working-class women.^49^ Interestingly, the miners’ trade union utilised women’s perspectives on care-giving in efforts to promote rehabilitation in the 1940s and wives of injured miners recounted the anxiety they endured as husbands and sons left each day for work, the shock suffered when the knock on the door signalled an injured man being carried from the pit, and the weeks or months of care provided in the home as the injured man’s condition improved. One woman, whose husband fractured his leg in an accident, noted how,

With the means at my disposal, along with the kind assistance of neighbours, I nursed and cared for my husband for eleven months in … crowded conditions. It was a hard and trying time as I had at this time five young children, the eldest of whom was nine years of age and the amount of compensation I received for this period was 24/- per week.

She also mentioned that her husband contracted pneumoconiosis later on in his working life and that he was ‘eight years at home’ before he died.^50^ It is perhaps unsurprising that an investigation of miners’ pulmonary disease in the period found that it was wives, rather than the men, who ‘remembered occasions and details of illnesses and death minutely, no doubt on account of their close association with the sickness’.^51^

As can clearly be seen, the extent to which disability impacted upon miners and their families could vary to a great degree, therefore, depending upon the interaction of a series of complex factors: the nature, severity and duration of a disabling injury; the size, composition and structure of the miners’ family; the specific place of the family within its life cycle when the disability occurred; and the number of disabled individuals within the family, as well as their particular positions within the family structure. In practice, what we see is that the actual lived experience of disability for mineworkers and their families was mediated as much by social factors as by purely pathological ones. This is an important point, which is worth emphasising: if disability is in some senses a societal construct, then we cannot hope to obtain a detailed understanding of the lived experience of being a person with a disability unless we are able to contextualise the lives of disabled individuals within their own social ecology framework.

## The social ecology of the family

A further level of complexity to be considered when analysing the impact of impairment on individuals and their families is the fact that they do not exist in isolation but instead operate within a broader context – this has been characterised by sociologists as the ‘social ecology framework’. Just as family systems theory can be used to structure studies of disability within the context of the family, so the social ecology model can assist efforts to place the family in its own particular contexts, consider the various influences that acted upon the family, and, crucially, give a better sense of the lived reality of disability in the past. In the social ecology model, there are different levels of interaction, both within and between families, communities and society more broadly. As Mishak, Seligman and Prezant observe, ‘To understand a family, it is not sufficient to study only certain family members. It is becoming increasingly important to examine the family in the context of larger social, economic and political realities’.^52^ Although the family may well have been the main locus of care for many impaired individuals in the south Wales coalfield in the chronological period considered here, there is nevertheless a continuous two-way interaction between an individual and his family on the one hand and the social ecology framework on the other, which manifests itself in a potentially infinite number of ways. In terms of the structure of how the social ecology model operates, Bronfenbrenner proposes that there are four principal levels: the microsystem, mesosystem, ecosystem, and macrosystem, with each system reflecting activity increasingly removed from the family (with a consequent diminishing of the ability of the impaired individual to exert a direct influence upon it) but nevertheless having an effect upon it.^53^ Within this model, the microsystem is defined as being the pattern of activities, roles, and interpersonal relations experienced by the family; the mesosystem is the wide range of settings in which a family actively participates, including medical and health care workers, extended family, friends and neighbours, work associates, other parents, and other local community factors; the ecosystem comprises remote settings that the family is not actively involved in yet can be affected by, such as mass media, health care systems, social welfare agencies, and educational systems; and the macrosystem is the overarching societal backdrop and its various socio-economic, political and cultural constituent elements.

These different systems will be taken in turn but, first, it is interesting to draw upon family systems theory to offer a theoretically-informed framework to consider the factors that led to and governed interactions between the family unit on the one hand and the various systems that existed beyond it on the other. Some families are characterised by permeable boundaries between individual family members whereby stress experienced by one member is easily transmitted to the other members of the family (such families are often described as ‘emotionally enmeshed’). This often coincides with rigid boundaries between the family and outside systems, and the family therefore tends to be isolated from the community and various agencies. In other instances, the emotional disengagement between family members can lead to more porous boundaries with the outside world that enable external systems, usually in the form of charities, local authorities and statutory welfare agencies, to enter the family system at ease and to impinge upon it without any difficulty.^54^ This is also complicated by families’ evaluation of the social and emotional costs of assistance from different providers of assistance. Working-class cultures of respectability, independence and self-sufficiency would have discouraged families from seeking assistance from the Poor Law or charities, though need would clearly have acted against such inclinations. On the other hand, communitarianism and mutualism would have facilitated interactions with such organisations as trade unions, friendly societies and workers’ medical schemes with fewer adverse consequences for the family’s standing in the community and perception of itself.^55^ The complex processes, considerations and motivations that influenced families’ deliberations on the decision to seek assistance are extremely difficult to observe, much less to understand, but historians must endeavour to explain these processes.

The microsystem within the social ecology model is of course broadly identical to the family unit described by family systems theory, the complexities of which were considered earlier in this essay. For this reason we do not propose to discuss it further here, other than to note that, for the south Wales coalfield in this period, the immediate family group was very much embedded in a broader mesosystem of extended family networks and close-knit communities built upon a high level of occupational homogeneity. This coalfield community mesosystem impacted upon the lived experience of impaired people in south Wales in innumerable ways, of course, with these patterns of interaction taking a range of forms, from spontaneous individual gestures to more organised communal and mutualist activities. At the one end of this scale we have an example from 1929 of an unmarried man from Llangeinor with an artificial leg: he is recorded as ‘residing with friends’ at that time, an arrangement presumably made because he had no other options open to him.^56^ A more coordinated but still essentially *ad hoc* response within the coalfield communities of south Wales to the plight of injured miners were workplace collections, which would have helped to lessen the blow of a reduced income or even to purchase an artificial limb. A slightly more sophisticated form of collections were ‘prize draws’, a type of ticket-based fundraising lottery for a draw to win prizes donated by individuals, groups and companies in the community. In 1901, for example, a prize draw was held in Llwydcoed, near Aberdare, in order to assist Jenkin Rees after he lost his arm in an accident at Abergorki Drift mine.^57^ Nor was assistance directed at financial help alone: Dick Cook, of Onllwyn in the Dulais Valley, remembered how, when he lost his sight, former work colleagues would call for him and ensure that he continued to socialise in order to cut down on the loneliness he would feel; he was even taken to rugby matches and friends would describe the action in the game to him.^58^

From a more institutional perspective, a prominent feature in the south Wales coalfield at this time was the miners’ union, the South Wales Miners’ Federation (SWMF, or simply ‘the Fed’), founded in 1898: this manifested itself at community level via its colliery workplace ‘lodges’. As Will Paynter (a lodge activist in the Rhondda valleys in the 1920s and later the general secretary of the National Union of Mineworkers) commented in his autobiography, ‘The Fed was a social institution and acted as such without question. Without doubt, its strength and ties with the communities was based on its intimate involvement in social and domestic affairs … The miners’ federation lodges were pillars of the communities’.^59^ The SWMF committed itself to taking an interest in all matters that affected the lives of its members and their families, acting as advocate and defender in a broad range of issues, and lodge officials acted as general counsellors to miners, their families and indeed other members of the community on a variety of different matters. One of the main areas where this manifested itself was the sheer volume of work undertaken by local lodge officials on behalf of the compensation cases of sick and impaired members. Other working-class institutions operated within the mesosystem, such a friendly societies, which paid relief monies to their members if they became injured or disabled, and medical aid societies, which were more comprehensive and provided a broad array of artificial limbs, surgical appliances and other ‘medical comforts’ to disabled workers and their family members.^60^ Such organisations ultimately belong within the mesosystem of the social ecology model, despite their size and structure, as they were an integral organic part of daily life in the coalfield communities of south Wales, were composed solely of members of those communities, and ultimately were democratic institutions which reflected the wishes and aspirations of the local populace, including disabled people. Unlike in other contexts, it would seem that solidarity continued to be maintained between disabled and other workers in south Wales, and the labour movement set out to represent the interests of injured or impaired miners in quite significant ways.^61^

Unlike organisations within the mesosystem, the ecosystem in the social ecology model comprises agencies beyond the control of individuals and their families yet with the ability to intervene in their lives directly and often with far-reaching consequences, whether positive or negative. For the south Wales coalfield at this time, this included local government authorities (primarily the various Boards of Guardians and their successors after the reform of state welfare provision in 1929), philanthropic organisations (such as the Swansea Hospital Ladies’ Samaritan Fund) and medical institutions (hospitals, orthopaedic clinics and convalescent homes). Disabled individuals could and did appeal to agencies within the ecosystem for assistance of various kinds, and it is evident from the sources that this was often provided – but the key point about this relationship is that it was these agencies which exercised control over welfare resources, retaining their prerogative over who should access them and to what degree. Significantly, it is organisations from this ecosystem stratum which were interested in investigating details of individuals’ family and financial circumstances, so as to better assess their eligibility for assistance – ironically, perhaps, this is why these kinds of records are one of the main types of evidence upon which this study has been based. With this in mind, historians should remember that such interactions between families on the one hand and organisations in the ecosystem on the other were often provoked by crises (‘traumatic stressors’ in the language of family systems theory) within the family unit that led that family to seek outside assistance rather than to continue to rely on its own resources and efforts; this also finds an echo in the crises observed by Cathy Smith that led families to admit mentally ill members of asylums.^62^

The patterns of interaction between an impaired individual and his/her social ecosystem reflected the complexity of his/her specific socio-economic, familial and pathological circumstances and the multiplicity of organisations and institutions operational within the south Wales coalfield at that time, some of which overlapped in function and purpose to varying degrees. Although it is impossible to describe this in detail, we can however sketch the principal characteristic features of a disabled individual’s engagement with his/her social ecosystem at this time. First, it should be noted that the evidence shows continuous and sometimes quite extensive interactions and negotiations between various ecosystem-level organisations and agencies regarding the support and assistance for a given impaired individual. This is an important factor to consider from the point of view of an individual’s access to the ‘mixed economy of care’, underlining the fact that the situation was never completely static but was always to some degree fluid and contingent. This process can be seen, for instance, in the example cited earlier of the woman from Loughor who required an artificial leg: her case was taken up by the Swansea Hospital Ladies’ Samaritan Fund between November 1928 and March 1929, whose representatives met with and also corresponded with the British Legion and two other ex-servicemen’s charities (as the woman’s husband was an ex-serviceman), with the result being that the three military funds ended up variously agreeing to provide sufficient funds to pay for her new artificial leg.^63^ Second, the provision of welfare and/or assistance for impaired individuals remained very much at the discretion of the relevant local government administrators and/or philanthropic institutions, based upon their assessments of the financial circumstances of the individual and his or her family. Inevitably, the outcome varied on a case-by-case basis. In the example mentioned earlier of the young girl from Cwmllynfell, for instance, the committee of the Swansea Hospital Ladies’ Samaritan Fund decided in November 1935 that it would meet the full cost of providing a glass eye for her.^64^ Conversely, in the case of the family from Blaengarw discussed earlier, the Bridgend and Cowbridge Poor Law Union Relief Advisory Committee eventually decided in December 1928 ‘that in view of the income to the home no assistance be granted’.^65^

Third, another noticeable factor is the extent to which it was necessary for impaired individuals and their families to engage with a whole range of different providers to obtain the required level of medical treatment and welfare support: hospitals, charities, local authorities, and so on. This can be seen in the case of the circumstances of a woman from Blaengarw and her family (a different family to the one cited above), who applied to the Swansea Hospital Ladies’ Samaritan Fund in February 1928 for a grant for train fares to attend hospital for her insulin treatment. The fund’s committee noted that the woman ‘has to attend at hospital at least once a month for diabetes treatment. It costs 8/- each time & her husband is very ill & only has 9/- Lloyd George money [that is, National Insurance statutory sick pay] & her eldest son an invalid for several years. The second earns £2.8 (of which 10/8 is kept back by Colliery Co. for rent) & the youngest is unemployed & gets 10/- dole’. It was agreed to pay her a grant of one pound, with a further grant of one pound being provided in two instalments for this purpose in December that year.^66^ Such complexity in the provision of assistance is notable and should perhaps indicate to historians that the idea of an ‘economy of makeshifts’ is as relevant to the twentieth century as the early modern period.^67^

Finally, notwithstanding all of the above complexities, the evidence shows that it was perfectly possible for impaired individuals to be completely dependent upon organisations and agencies external to the family unit. One clear example of how this ‘mixed economy of care’ could operate is provided by the case noted in September 1926 of a sixty-seven year old colliery haulier and his wife: his wife was paralysed, they had no children dependent or contributing to the household’s finances, and he had been out of work since August 1925. In these circumstances, the entirety of their income consisted of relief payments from Bedwellty Poor Law Union (inclusive of a supplement on account of his wife’s medical requirements), his National Insurance unemployment benefit and a smaller amount from a friendly society.^68^ It seems apparent that, despite the contingent and negotiated nature of provision and depending upon individual circumstances, this array of fairly remote ecosystem-level organisations and institutions could indeed collectively constitute a significant, if rather piecemeal, welfare network for impaired individuals.

From the perspective of attempting to apply the social ecology model to a particular set of chronological and geographical circumstances, it is in the macrosystem that the main difference between the theory’s sociological origins and its application to a specific historical context becomes apparent. As with most sociological concepts, this model was conceptualised in essentially present-day terms; here, the macrosystem functions essentially as a static socio-cultural backdrop. In the historical usage of the social ecology framework that we have been outlining, however, the macrosystem is a dynamic environment, capable of generating dramatic changes which could have profound and life-altering consequences for impaired individuals and their families. We believe that this difference is certainly not a failing and, if anything, it helps the historical application of the social ecology model to attain a greater degree of explanatory power, by demonstrating that the lived experience of impaired individuals and their families was not just mediated by their immediate environment but was also intimately connected to and bound up with the broadest socio-economic, political and cultural trends within society as a whole.

For the south Wales coalfield and its inhabitants, by far the most significant development within the macrosystem in the interwar period was of course the dramatic downturn in the fortunes of the coal industry. This process occurred as the consequence of a variety of factors specific to the industry, although for exporting coalfields like south Wales the whole phenomenon was exacerbated markedly by broader international economic developments, particularly the effect of the Great Depression.^69^ This can be seen obviously in the many tens of thousands who were condemned to a period of long-term unemployment or else never worked again. Disabled miners were certainly not spared this fate. One example of this is that of a miner from Penllergaer near Swansea, who had lost his leg following a colliery accident and undergone an operation in Swansea Hospital in 1922; he received compensation and found employment doing light work at the colliery, although subsequently lost his job following the miners’ lockout of 1926 and by 1930 had been continuously unemployed since then.^70^ In this instance, we see one way in which the successful attempt by an impaired individual to return to employment following his disablement could be undone as a direct consequence of macrosystem-level occurrences. These developments could also manifest themselves via a more indirect chain of causality: to take one case cited earlier, the Nantyglo miner-turned-confectioner’s business failed in 1922, due primarily to the impact upon the disposable income of his clientele of the coal industry’s decline after 1921.^71^

In addition to general economic trends, specific political and legislative developments in the macrosystem could also have a direct bearing upon the lives of disabled individuals. One obvious political example of this is the impact of the decision taken in 1925 by the Chancellor of the Exchequer, Winston Churchill, to return sterling to the Gold Standard. As Morgan notes, ‘the outcome was a disaster for British coal exports which were now seriously over-valued in relation to foreign currency, and was directly responsible for increasing unemployment and further decline in the Welsh mining industry’.^72^ Conversely, the advent of legislation such as the Workmen’s Compensation Act of 1897 established a statutory system of compensation for industrial injury and disability, thereby helping to provide a degree of financial stability and security for many tens of thousands of disabled mineworkers. This statutory system was amended on a number of occasions in the years and decades after 1897, most notably in the interwar period with the addition of ‘silicosis’ to the list of compensatable diseases in 1928 and an extension of this part of the legislation to a larger group of the mining workforce in 1934; such extensions and amendments were the difference between receiving and not receiving compensation.^73^

Furthermore, developments within the macrosystem could also directly and significantly affect the extent to which community-based mesosystem organisations were able to play a role within the lives of disabled individuals. After the defeat of the miners in the lockout which followed the General Strike of 1926, for instance, the increased industrial relations power of the coalowners, the onset of large-scale unemployment and also a degree of disillusionment with the union meant the membership of the SWMF collapsed during 1927, from 136,250 to 72,981 – with obvious consequences for the ability of the Fed to assist disabled mineworkers.^74^ The SWMF subsequently spent most of the 1930s in a gradual struggle to re-establish its prominent role within coalfield communities.^75^ Crucially, therefore, the ability of the miners’ trade union to defend their impaired members and secure benefits that would have ameliorated the extent of disability experienced by the individual and by the family was lessened from 1926 until the late 1930s as a result of the industrial context. Finally, it should also be noted that even the social ecosystem stratum was not immutable and could sometimes be altered by macrosystem-level events: although it falls slightly outside the timeframe considered in this essay, for our purposes the best and most prominent example of this was the establishment of the National Health Service in 1948.

## Conclusion

Family systems theory and the social ecology approach to the family have not been without their critics and it is clear that many of the criticisms are as relevant to historical as to contemporary uses of the theories. As with most sociological theories, their presentism stands in contrast to the historian’s interest in change over time. While therapists who utilise family systems theory in their work take account of change within any family they treat, broader social and cultural changes over longer periods of time, many of which change the very character and definition of the family, are not accounted for in ways that historians would value within these theories. This is especially true of the focus on the ‘nuclear family’ in these theories that conceptualises the constitution of the family as including two parents and any children. Even if that definition of the family was not without its problems in the present, it becomes even more problematic in the past when household composition was far more varied than this would suggest – the cases for interwar south Wales cited above demonstrate this complexity. Moreover, family systems theory, in its application in therapy and social work in relation to disability, tends to give attention to families in which a child experiences an impairment and is perhaps less attuned to instances, such as those outlined in this industrial context, in which adults suffer disability or where there are a number of family members with impairments.^76^ Most significantly of all, critiques of family systems theory noted its particular cultural dimensions and its inappropriateness for cultures other than those in which it was rooted. Questions have been raised about the extent to which it takes for granted ideas of class, gender, ethnicity and race, and concerns have been raised about the theory’s applicability in light of the extent of human and familial variability. Historians, it is clear, would need to be attuned to the particular cultural contexts in which they utilise the theory.^77^

It is clear, nevertheless, that a sociologically-informed perspective can help to bring an extra level of precision to the analysis of disability in any particular historical context. This case study of south Wales coalfield society in the interwar period demonstrates the ways in which family systems theory and the social ecology model of disability can aid attempts to understand how impairment in the past affected families as much as the individual family members who were disabled, and how the experience of disability in each individual case was impacted upon and shaped in a variety of different ways by an interconnected and interacting hierarchy of social networks: from dynamics within the family, through community-based factors, to the broader socio-economic and political context of the time. Through the use of these theories, historians are aided in their attempts to historicise impairment and locate it in the particular contexts of disabled people’s lives. They help to situate the impaired person in the context that was most important to them, the family, and allows us to get a much better sense of the lived experience of disability as it was experienced by disabled people and their families.

## Notes

1 L. E. Mishak, M. Seligman and F. Prezant, *Disability and the Family Life Cycle* (New York: Basic Books, 1999), 1.

2 D.V. Papero, *Bowen Family Systems Theory* (MA: Allyn and Bacon, 1999); M.M. Dore, ‘Family Systems Theory’, in *Comprehensive Handbook of Social Work and Social Welfare: Volume 2. Human Behaviour in the Social Environment*, ed. B.A Thyer (New Jersey: John Wiley & Sons, 2008), 431–62.

3 Mishak, Seligman and Prezant, *Disability and the Family Life Cycle*, 9–10.

4 E.K. Cridland et al., ‘Family-focused Autism Spectrum Disorder Research: A Review of the Utility of Family Systems Approaches’, *Autism*, 18, no. 3 (2014), 213–22; A. Zaidman-Zait et al., ‘The Impact of Childhood Hearing Loss on the Family: Mothers’ and Fathers’ Stress and Coping Resources’, *Journal of Deaf Studies and Deaf Education*, 21, no. 1 (2016): 23–33.

5 P. Horden and R. Smith, ed., *The Locus of Care: Families, Communities, Institutions and the Provision of Welfare Since Antiquity* (London: Routledge, 1998).

6 D. Wright, ‘Familial Care of “Idiot Children” in Victorian England’, in *The Locus of Care*, eds. Horden and Smith, 176–97; C. Smith, ‘Living with Insanity: Narratives of Poverty, Pauperism and Sickness in Asylum Records 1840–76’, in *Poverty and Sickness in Modern Europe: Narratives of the Sick Poor, 1780*–1938, ed. A. Gestrich, E. Hurren and S. King (London: Continuum, 2012), 117–41; S. Taylor, ‘“All his Ways are Those of an Idiot”: The Admission, Treatment of and Social Reaction to Two “Idiot” Children of the Northampton Pauper Lunatic Asylum, 1877–1883’, *Family and Community History*, 15, no. 1 (2012): 34–43.

7 M. Katz and C. Sachße, eds., *The Mixed Economy of Social Welfare: Public/private relations in England, Germany and the United States, the 1870s to the 1930s* (Baden Baden: Nomos, 1996); N. Johnson, *Mixed Economies of Welfare: A Comparative Perspective* (Hemel Hempstead: Prentice Hall Europe, 1999); S. Thompson, ‘The Mixed Economy of Care in the South Wales Coalfield, c.1850–1950’, in *Healthcare in Ireland and Britain from 1850: Voluntary, Regional and Comparative Perspectives*, eds. D. S. Lucey and V. Crossman (London: Institute of Historical Research, 2015).

8 J. Lewis, ‘Gender, the Family and Women’s Agency in the Building of “Welfare States”: The British Case’, *Social History*, 19, no. 1 (1994): 38.

9 Horden and Smith, *The Locus of Care*, 9.

10 A. McIvor and R. Johnston, *Miners’ Lung: A History of Dust Disease in British Coal Mining* (Aldershot: Ashgate, 2007), 45.

11 J. Benson, *British Coalminers in the Nineteenth Century: A Social History* (Dublin: Gill and Macmillan, 1989), 43.

12 D. Jones, ‘Counting the Cost of Coal: Women’s Lives in the Rhondda, 1881–1911’, in *Our Mother’s Land: Chapters in Welsh Women’s History, 1830*–1939, ed. Angela V. John (Cardiff: University of Wales Press, 1991), 109.

13 R. Church, *The History of the British Coal Industry, Vol. 3, 1830*–*1913: Victorian Pre*-*eminence* (Oxford: Clarendon Press, 1986), 588.

14 J. Williams, *Digest of Welsh Historical Statistics, Vol. 1* (Cardiff: Welsh Office, 1985), 332; Church, *The History of the British Coal Industry*, 189.

15 Mishak, Seligman and Prezant, *Disability and the Family Life Cycle*, 1.

16 Mishak, Seligman and Prezant, *Disability and the Family Life Cycle*, 8.

17 Benson, *British Coalminers in the Nineteenth Century*, 121.

18 M. R. Haines, ‘Fertility, Nuptiality, and Occupation: A Study of Coal Mining Populations and Regions in England and Wales in the Mid-Nineteenth Century’, *Journal of Interdisciplinary History*, 8, no. 2 (1977): 259–62.

19 D. Beddoe, *Out of the Shadows: A History of Women in Twentieth*-*Century Wales* (Cardiff: University of Wales Press, 2000); A.V. John, *Our Mother’s Land: Chapters in Welsh Women’s History, 1830*–*1939* (Cardiff: University of Wales Press, 1991).

20 J. Davies, *A History of Wales* (London: Penguin, 2007), 588.

21 Glamorgan Archives, UM/30/16, Merthyr Tydfil Union, Workhouse admission register, July 1929.

22 Benson, *British Coalminers in the Nineteenth Century*, 113.

23 Gwent Archives, CSWBGB/C/29, Bedwellty Union Out Relief Advisory Committee (BUORAC) Papers, 18 February 1924.

24 Gwent Archives, CSWBGB/C/32, BUORAC Papers, 29 December 1926.

25 Gwent Archives, CSWBGB/C/30, BUORAC Papers, 1925 and 1926.

26 BUORAC Papers, Advisory Committee papers, 10 December 1923.

27 R. Anderson and M. Bury, eds., *Living With Chronic Illness: The Experience of Patients and Their Families* (London: Unwin Hyman, 1988).

28 South Wales Miners’ Library, Swansea University, AUD/164, John Morgan Evans oral history interview.

29 B. Curtis and S. Thompson, ‘“A Plentiful Crop of Cripples Made by All This Progress”: Disability, Artificial Limbs and Working-class Mutualism in the South Wales Coalfield, 1890–1948’, *Social History of Medicine*, 27, no. 4 (2014): 715; M. Mantin, ‘“From Pithead to Sickbed” Exhibition Blogs: George Preece, Disabled Miner’, Disability and Industrial Society website, http://www.dis-ind-soc.org.uk/en/blog.htm?id=45, 2015.

30 South Wales Miners’ Library, Swansea University, AUD/382, D.C. Davies oral history interview.

31 B.L. Coombes, *Miners Day* (Harmondsworth: Penguin Books, 1945), 113.

32 Papero, *Bowen Family Systems Theory*, 37–65; Dore, ‘Family Systems Theory’, 442.

33 Smith, ‘Living with Insanity’, 136.

34 BUORAC Papers, Advisory Committee papers, 17 December 1923, Advisory Cases, No.6 District.

35 Glamorgan Archives, UB/7/1, Bridgend and Cowbridge Relief Committee (BCRAC) minutes, 8 June 1929.

36 West Glamorgan Archive Service, D/D SHF/3, Swansea Hospital Ladies’ Samaritan Fund (SHLSF) minutes, 10 May 1935.

37 SHLSF minutes, 8 November 1935.

38 BUORAC Papers, 18/20 October 1926.

39 Glamorgan Archives, UB/6, BCRAC minutes, 5 December 1928.

40 Mishak, Seligman and Prezant, *Disability and the Family Life Cycle*, 8.

41 S. Bruley, ‘“Little Mothers”: Adolescent Girls and Young Women in the South Wales Valleys between the Wars’, *Llafur*, 10, no. 3 (2010): 126–44.

42 BUORAC Papers, 18/20 October 1926.

43 Dore, ‘Family Systems Theory’, 448.

44 E.L. Collis and Major Greenwood, *The Health of the Industrial Worker* (London: Churchill, 1921), 409.

45 C. White and S.R. Williams, *Struggle or Starve: Women’s Lives in the South Wales Valleys between the Two World Wars* (Dinas Powys: Honno Press, 1998), 15.

46 SHLSF minutes, 12 May 1911.

47 SHLSF minutes, 9 November 1928.

48 SHLSF minutes, 12 February 1926.

49 N. M. Forestell, ‘“And I Feel Like I’m Dying from Mining for Gold”: Disability, Gender, and the Mining Community, 1920–1950’, *Labor: Studies in Working*-*Class History of the Americas*, 3, no. 3 (2006): 83; M. Ll. Davies, *Life As We Have Known It* (London: J. and A. Churchill, 1931); M. Spring Rice, *Working*-*class Wives: Their Health and Conditions* (Harmondsworth: Penguin Books, 1939).

50
*The Miner*, August 1946, 7.

51 E.M. Williams, *The Health of Old and Retired Miners in South Wales* (Cardiff: University of Wales Press Board, 1933), 22.

52 Mishak, Seligman and Prezant, *Disability and the Family Life Cycle*, 10.

53 U. Bronfenbrenner, *The Ecology of Human Development* (Cambridge, MA: Harvard University Press, 1979).

54 Dore, ‘Family Systems Theory’, 448–9.

55 E. Hopkins, *Working*-*class Self*-*help in Nineteenth Century England: Responses to Industrialisation* (London: UCL Press, 1995); P. Johnson, *Saving and Spending: The Working*-*class Economy in Britain 1870*–*1939* (Oxford: Clarendon. 1985).

56 BCRAC minutes, 3 July 1929.

57
*Tarian y Gweithiwr*, 2 May 1901.

58 South Wales Miners’ Library, Swansea University, AUD/222, Dick Cook oral history interview.

59 W. Paynter, *My Generation* (London: Allen & Unwin, 1972), 110–11.

60 Curtis and Thompson, ‘A Plentiful Crop of Cripples Made by All This Progress’.

61 Curtis and Thompson, ‘A Plentiful Crop of Cripples Made by All This Progress’; Forestell, ‘And I Feel Like I’m Dying from Mining for Gold’, 80; in contrast, see J. Williams-Searle, ‘Cold Charity: Manhood, Brotherhood, and the Transformation of Disability, 1870–1900’, in *The New Disability History: American* Perspectives, eds. P. Longmore and L. Umansky (New York, 2001), 157–86.

62 Dore, ‘Family Systems Theory’, 442; Smith, ‘Living with Insanity’.

63 SHLSF minutes, 9 November, 14 December 1928, 11 January, 9 February, 8 March 1929.

64 SHLSF minutes, 8 November 1935.

65 SHSLF minutes, 5 December 1928.

66 SHLSF minutes, 18 February, 9 March, 14 December 1928.

67 S. King and A. Tomkins, eds., *The Poor in England, 1700*–*1850: An economy of makeshifts* (Manchester: Manchester University Press, 2003).

68 BUORAC papers, 1/6 September 1926.

69 Supple, *The History of the British Coal Industry, Vol. 4, 1913*–*1946*, 271–2.

70 SHLSF minutes, 14 March 1930.

71 BUORAC Papers, 10 December 1923.

72 K. O. Morgan, *Rebirth of a Nation: Wales 1880*–*1980* (Oxford: Oxford University Press, 1981), 213.

73 M. Bufton and J. Melling, ‘“A Mere Matter of Rock”: Organized Labour, Scientific Evidence and British Government Schemes for Compensation of Silicosis and Pneumoconiosis among Coalminers, 1926–1940’, *Medical History*, 49, no. 2 (2005): 161–3.

74 C. Williams, *Capitalism, Community and Conflict: The South Wales Coalfield, 1898*–*1947* (Cardiff: University of Wales Press, 1998), 89.

75 H. Francis and D. Smith, *The Fed: A History of the South Wales Miners in the Twentieth Century* (London: Lawrence & Wishart, 1980).

76 Cridland et al., ‘Family-focused Autism Spectrum Disorder Research’; Zaidman-Zait et al., ‘The Impact of Childhood Hearing Loss on the Family’.

77 Dore, ‘Family Systems Theory’, 444, 456–7; Mishak, Seligman and Prezant, *Disability and the Family Life Cycle*, 3–4.

